# A Presentiment

**Published:** 1856-02

**Authors:** 


### A PRESENTIMENT,

Is an impression on the mind, that *something is going to take place*, and usually such is the case; perhaps we may say without exaggeration, that something always does occur, after a presentiment is formed; if such were not the fact, we cannot conjecture what would become of every body. Just imagine for a moment, that something did not take place in such a large world as this!

Presentiments love weak places, hence they flourish among weak-minded people, not necessarily weak-minded by nature, but made so by a diseased body. We are told of a young lady at Kinderhook, who was visited by an apparition two years ago, at dead hour of night, which announced to her in solemn accents, that in two years she would be the inhabitant of another and a better world; this circumstance had such a depressing influence on her mind, that she pined away by degrees and did die, at the close of the term named, and was buried a few days ago.

An eminent clergyman, on parting from another in St. Louis, said: "I have a strong presentiment that we shall never meet again," and within a few hours he perished at the Gasconade on the Pacific Railway.

An almost infallible cure for presentiment, however violent, is a good emetic, a grubbing hoe, with a few days bread and water diet. For ourselves, we would omit the emetic, as we do not patronize physic, except by proxy. The reason we give medicine at all is that people are always in a hurry, not exactly to get well, but to get able to eat; if they can only eat, nine out of ten think they are getting along famously. Every body wants to get well in a minute, and for the bare chances of doing so, with a slight degree of assurance to that effect from any knave who is willing to promise, it having the wit to see at a glance that the assurance must be father to the fee—we repeat, with a very slim assurance of being made well in a short time, the large majority of invalids would swallow a quart of Shakespeare's soup thrice a day, said soup being made, as the reader may remember, by several old witches, of such things as newt's eyes, frog's toes, lizzard wings, stings of rattle-snakes and other ingredients not necessary to be named, but all brought to the climatic point by—onions.

An emetic will dissipate a presentiment in five minutes, while the vigorous use of the grubbing hoe in the open air, would work off the extra and thick blood, abuse accumulation in the brain generates these diseased imaginings, while the diet of bread and water would supply a pure article of blood in the place of the impure material.

Whoever heard of a healthy, out door, day laborer, having a "Presentiment" in the pursuit of his occupation? The fact is, they have not time to be moping about such tom-fooleries; the only presentiment that ever troubles them is a veritable fact, a tangible reality. "Root Pig, or Die" is their ever living ghost.

Presentiments do not exist except in connection with one of the three following things—1. A weak mind. 2d. A diseased body. 3d. An idle condition of life.

Loafing and gluttony are the great originators of this unfortunate condition of mind and its almost certain removal follow in temperate eating, combined with physical activity. If unattended to, and friendly death does not step in to save from a greater calamity, insanity winds up the history.

To the reflecting, we suggest a fact which dissipates the mystery which hangs around "Presentiments." In ordinary cases, a thing is not baptized as a "Presentiment," until the coincidence of the fact. Superstitious minds, in which presentiments mostly dwell, take no note of the countless impressions that certain things might take place, which did not afterwards take place; one such coincidence makes an impression against a million non-concurrents.

